# Liver PDFF estimation using a multi-decoder water-fat separation neural network with a reduced number of echoes

**DOI:** 10.1007/s00330-023-09576-2

**Published:** 2023-04-04

**Authors:** Juan Pablo Meneses, Cristobal Arrieta, Gabriel della Maggiora, Cecilia Besa, Jesús Urbina, Marco Arrese, Juan Cristóbal Gana, Jose E. Galgani, Cristian Tejos, Sergio Uribe

**Affiliations:** 1 Biomedical Imaging Center, Pontificia Universidad Católica de Chile, Santiago, Chile; 2 Millennium Institute for Intelligent Healthcare Engineering iHEALTH, Santiago, Chile; 3 Department of Electrical Engineering, Pontificia Universidad Católica de Chile, Santiago, Chile; 4 Faculty of Engineering, Universidad Alberto Hurtado, Santiago, Chile; 5 Department of Radiology, School of Medicine, Pontificia Universidad Catolica de Chile, Santiago, Chile; 6 Complejo Asistencial Dr. Sótero del Río, Santiago, Chile; 7 Department of Gastroenterology, School of Medicine, Pontificia Universidad Católica de Chile, Santiago, Chile; 8 Department of Pediatric Gastroenterology and Nutrition, Division of Pediatrics, School of Medicine, Pontificia Universidad Católica de Chile, Santiago, Chile; 9 Department of Health Sciences, Nutrition and Dietetics Career, Faculty of Medicine, Pontificia Universidad Católica de Chile, Santiago, Chile; 10 Department of Nutrition, Diabetes and Metabolism, Faculty of Medicine, Pontificia Universidad Católica de Chile, Santiago, Chile; 11 Department of Medical Imaging and Radiation Sciences, School of Primary and Allied Health Care, Faculty of Medicine, Nursing and Health Sciences, Monash University, Melbourne, Australia

**Keywords:** Liver, Non-alcoholic fatty Liver disease, Biomarkers, Deep leaning, Magnetic resonance imaging

## Abstract

**Objective:**

To accurately estimate liver *PDFF* from chemical shift-encoded (CSE) MRI using a deep learning (DL)-based Multi-Decoder Water-Fat separation Network (MDWF-Net), that operates over complex-valued CSE-MR images with only 3 echoes.

**Methods:**

The proposed MDWF-Net and a U-Net model were independently trained using the first 3 echoes of MRI data from 134 subjects, acquired with conventional 6-echoes abdomen protocol at 1.5 T. Resulting models were then evaluated using unseen CSE-MR images obtained from 14 subjects that were acquired with a 3-echoes CSE-MR pulse sequence with a shorter duration compared to the standard protocol. Resulting PDFF maps were qualitatively assessed by two radiologists, and quantitatively assessed at two corresponding liver ROIs, using Bland Altman and regression analysis for mean values, and ANOVA testing for standard deviation (STD) (significance level: .05). A 6-echo graph cut was considered ground truth.

**Results:**

Assessment of radiologists demonstrated that, unlike U-Net, MDWF-Net had a similar quality to the ground truth, despite it considered half of the information. Regarding *PDFF* mean values at ROIs, MDWF-Net showed a better agreement with ground truth (regression slope = 0.94, *R*^2^ = 0.97) than U-Net (regression slope = 0.86, *R*^2^ = 0.93). Moreover, ANOVA post hoc analysis of STDs showed a statistical difference between graph cuts and U-Net (*p* < .05), unlike MDWF-Net (*p* = .53).

**Conclusion:**

MDWF-Net showed a liver *PDFF* accuracy comparable to the reference graph cut method, using only 3 echoes and thus allowing a reduction in the acquisition times.

**Clinical relevance statement:**

We have prospectively validated that the use of a multi-decoder convolutional neural network to estimate liver proton density fat fraction allows a significant reduction in MR scan time by reducing the number of echoes required by 50%.

**Key Points:**

• *Novel water-fat separation neural network allows for liver PDFF estimation by using multi-echo MR images with a reduced number of echoes*.

• *Prospective single-center validation demonstrated that echo reduction leads to a significant shortening of the scan time, compared to standard 6-echo acquisition*.

• *Qualitative and quantitative performance of the proposed method showed no significant differences in PDFF estimation with respect to the reference technique*.

**Supplementary Information:**

The online version contains supplementary material available at 10.1007/s00330-023-09576-2.

## Introduction

Non-alcoholic fatty liver disease (NAFLD) is a spectrum of disorders, that range from simple steatosis to non-alcoholic steatohepatitis and cirrhosis [1]. NAFLD is directly related to the hepatic fat content [1, 2] and, despite biopsy being the reference method to estimate liver fat, non-invasive modalities based on chemical shift-encoded (CSE) MRI have been increasingly used. This procedure allows the estimation of proton density fat fraction (*PDFF*), which has already been validated against pathology to determine the presence and grading of hepatic steatosis in patients with NAFLD [1].

To accurately estimate *PDFF*, the standardized techniques must address a water-fat separation problem considering the effect of a set of confounders, such as R2* (= 1/T2*) signal decay ratio, off-resonance field map (Δ*f*), and multi-peak fat spectrum, as they have non-linear effects over the signal that complicate its separation [3–5].

Several water-fat separation algorithms that deal with these confounding factors have already been proposed [3, 6–8]. A graph cut–based method, that consists of a VARPRO formulation and an iterative Graph-Cut algorithm of considerable robustness and accuracy, is usually considered the gold standard [8, 9]. However, the required CSE-MR images, which are acquired using a multiple gradient echo pulse sequence, are usually obtained with 6 echoes, as this is the minimum recommended number in literature to achieve an improved R2* estimation [10]. Therefore, scan times could be significantly long and several patient breath holdings could be necessary during the procedure. Moreover, The graph cut method requires large computational resources and calculation time.

Recently, several deep learning (DL)–based methods have been proposed to address the water-fat separation problem [11–15]. To our knowledge, all these works have proposed convolutional neural networks (CNNs) with different configurations, although most of them [11, 12, 15] have implemented a U-Net architecture [16]. However, only one of them has included the estimation of R2* and Δ*f* maps to discard them as confounding factors [15]. Moreover, only a couple of these works have performed an assessment using images with a reduced number of echoes [11, 12], which could suggest a possible reduction in the acquisition times. Nevertheless, the validation of reducing the number of echoes has not been thoroughly done, as the performance was not assessed using acquisitions of a shorter duration than the original protocol.

To achieve an effective shortening of the scan time, the repetition time (TR) of the pulse sequence must be optimized to maximize the acquired number of slices per breath-hold. Shortening of TR is known to generate a T1-weighting in the resulting MR signal and, as consequence, a positive *PDFF* bias. To avoid this bias, low flip angles are preferred at the cost of a drop in the signal intensity [17].

Recent DL applications for signal processing have implemented multi-task U-Net-based architectures to jointly perform more than one estimation from a single input [18, 19]. This configuration, which is a U-Net with multiple decoders, allows a more dedicated estimation of outputs with significantly different attributes (i.e., sharpness of structures, noise, etc.) without the need of developing two separate models for each task.

In this work, we propose a multi-task U-Net-based architecture, denoted as a multi-decoder water-fat separation neural network (MDWF-Net), to jointly estimate water-only and fat-only images, in addition to R2* and Δ*f* maps. This multi-task approach allows for improving the estimation of water-fat images, which enables a reduction of the necessary echoes to achieve an accurate *PDFF* quantification. The main goal of this work was to achieve a liver *PDFF* estimation with an accuracy comparable to the reference 6-echo graph cut technique, using 3-echo images.

## Materials and methods

The data for this study was acquired in a single center, with a 1.5-T MR scanner (Achieva, Philips Healthcare), and was HIPAA compliant.

### Retrospective data

We first considered a set of previously collected CSE-MR abdomen images, which were obtained between 2017 and 2020. Data was obtained from 134 volunteers, each of them with a number of slices between 18 and 24, according to the whole liver coverage. Both healthy and fatty-liver subjects were considered. For non-healthy subjects, the inclusion criterion was suspicion of hepatic steatosis preliminarily evaluated by ultrasound. Additionally, fatty-liver subjects were considered only after discarding confounding conditions (i.e., history of other chronic liver diseases, significant alcohol consumption, and drug therapy).

These CSE-MR images were acquired with a standard 6-echo T1w-GRE protocol. During acquisition, patients were asked for breath-holding (10–13 breath-holds of approximately 10 s each), and complex-valued data was stored. The most relevant pulse sequence parameters were the following: TR = 30 [ms], TE_1_/ΔTE = 1.3/2.1 [ms], voxel-size = 0.89 × 0.89 × 10 [mm^3^], flip-angle = 10°. The remaining parameters are summarized in Table 1.

**Table 1:** MR scan acquisition parameters of the used pulse sequences. (*) Repetition time (TR) was set as “shortest”; this stablished TR values that varied according to the breath-hold duration

Acquisition	6-echoes GRE	3-echoes GRE
Total number of echoes	6	3
Echo train length	6	3
TE_1_ / ΔTE (ms)	1.29 / 2.1	1.29 / 2.1
TR (ms)	30	13–47 (*)
Flip angle	10°	10°
NSA	2	2
Slice thickness (mm)	10	10
Nominal scan time (s)	120	37–54
Pixel bandwidth (Hz)	1736–1786	1736–1786
Phase encoding steps	230	230
Frequency encoding steps	232	232

### Prospectively acquired images

We scanned a second subset of healthy subjects (between 2021 and 2022) using the standard 6-echoes protocol and a 3-echoes acquisition. The 3-echoes pulse sequence considered the same echo times of the first 3 echoes of standard protocol, except for TR, which was optimized at each scan to maximize the number of acquired slices per breath-hold, leading to a reduction of the nominal scan time from 120 to 54 s. Pulse sequence parameters of the 3-echoes protocol are also summarized in Table 1.

### Reference water-fat separation method

The voxel-wise physical model that describes the complex signal at the *n*-th echo (*I*_*n*_) for a T1w-GRE sequence is:

1 $$I_n\left(PW,PF,R_2^\ast,\Delta f\right)=e^{-R_2^\ast\cdot TE_n}\cdot e^{j2\pi\Delta f\bullet{TE}_n}\cdot\left[PW+PF\sum_{p=1}^P\alpha_p\cdot e^{j2\pi f_{F,p}\cdot{TE}_n}\right]$$

where *j* is the imaginary unit, *ρ*_*w*_ and *ρ*_*f*_ are the water and fat signals, R2* is the transverse relaxation, Δ*f* is the off-resonance field map, *TE*_*n*_ is the *n*-th echo time, *P* is the number of fat spectral peaks, and *f*_*F,p*_ are the frequencies of the fat spectral peaks, with their respective relative amplitudes *α*_*p*_ (with *Σα*_*p*_ = 1), which are known a priori [20]. Then, *PDFF* is calculated as:

2 $$PDFF=\frac{\left|PF\right|}{\left|PW\right|+\left|PF\right|}$$

Reference results of *ρ*_*w*_, *ρ*_*f*_, R2*, and Δ*f* were obtained using the graph cut algorithm [8] implemented in the International Society of Magnetic Resonance in Medicine (ISMRM) water-fat Toolbox[21].

### Multi-decoder water-fat separation network

**Fig. 1:**
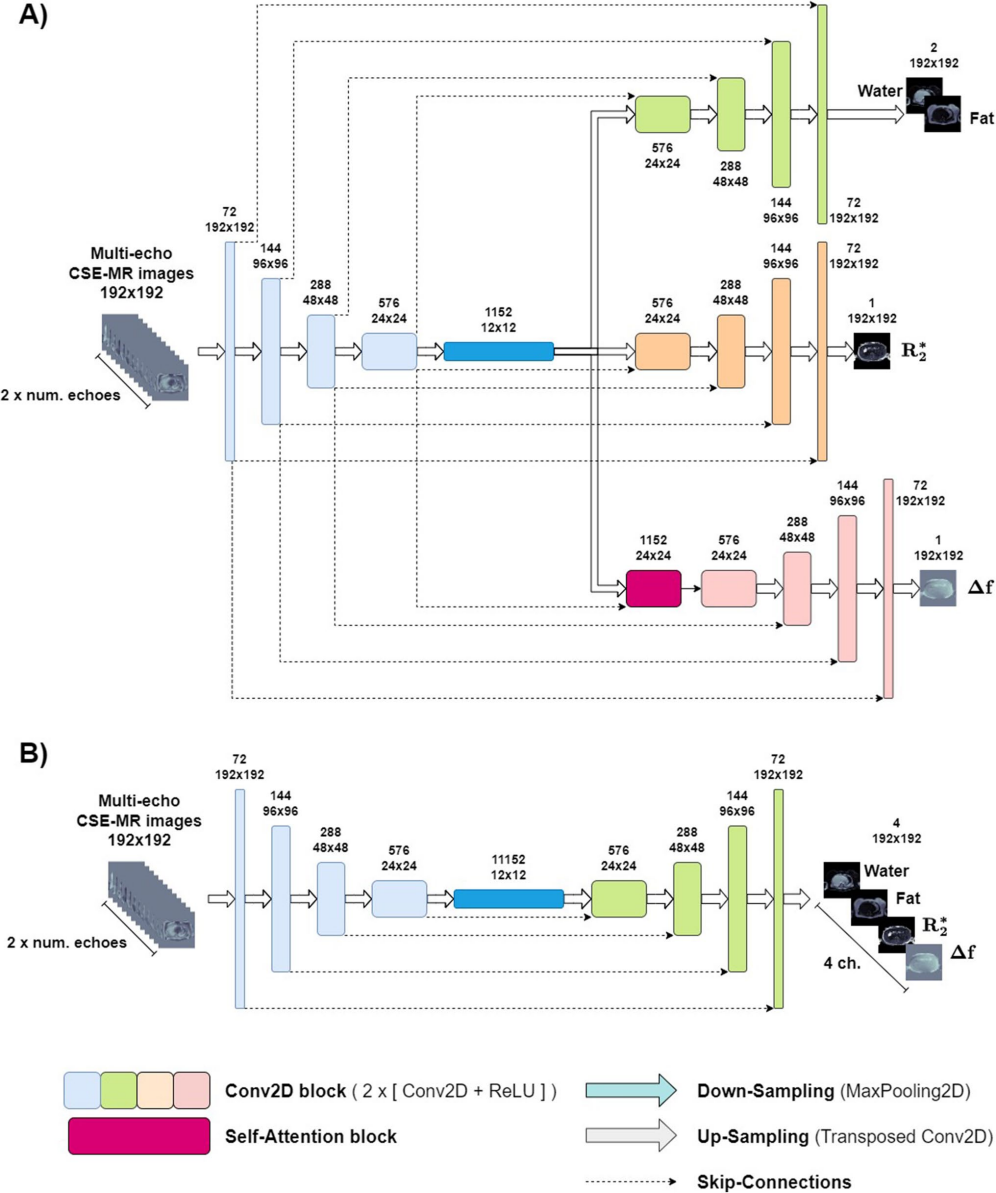
**A** Architecture of MDWF-Net. An encoder structure (light blue) extract features from the multi-echo acquisitions, converging to the reduced-dimensions latent space (blue). From this latent space, three decoders translate these extracted features into water-fat images (green), R2* map (orange), and off-resonance field map (pink). **B** A similar U-Net architecture, with a single decoder to obtain the four outputs from the latent space

MDWF-Net is a CNN [22, 23] capable of calculating water-fat images, R2* and ∆*f* after receiving multi-echo GRE acquisitions as input. The architecture of MDWF-Net is based on multi-task U-Net, which has been previously proposed in the literature for signal processing tasks [18, 19]. This configuration consists of an encoder-decoder structure that translates the input to a reduced-dimensions latent space of features, from which water-fat images, R2* and ∆*f* are separately decodified (Fig. 1).

The encoder was comprised of 4 consecutive Conv2D blocks, which were composed of two bidimensional convolution operators (with *N* = 72, 144, 288, 576 convolutional filters) each followed by ReLU activation function and Batch Normalization. The resulting Conv2D blocks features were downsampled using 2D max pooling, to finally obtain the latent space, which consisted of 1152 features of 12 × 12 dimensions. In the decoders, latent space features were upsampled using transposed bidimensional convolutions and then concatenated with the equally shaped encoder features. The concatenated features were then processed through the same Conv2D blocks used in the encoder. For Δ*f* decoder, the first upsampling from the latent space was followed by the application of a self-attention block [24–26] that enabled the learning of spatial relationships between non-neighboring pixels. This additional self-attention block was expected to improve the suppression of swapping artifacts. Therefore, we expected MDWF-Net to overperform the already studied U-Net [11, 12, 15] (Fig. 1B), as it gives a more dedicated approach to the specific features of each output.

The input for MDWF-Net was arrays composed of multiple echoes of a single rescaled 2D slice of the abdomen. All CSE-MR images were rescaled to 192 × 192 by k-space subsampling, and each array channel contained the real and imaginary components of each echo. Therefore, for 3-echoes acquisitions, the input array dimensions were 192 × 192 × 6. On the other hand, MDWF-Net returned three outputs: a 2-channels array with the magnitude water-fat images, and two single-channel arrays with the R2* and the Δ*f* maps, respectively.

### Experiments

Previously acquired data were considered for training (*N* = 2479 slices) and validation during training (*N* = 384), while prospective data were reserved for testing. As previous data included 6-echoes images, and it would have been non-viable to re-scan each of the previously acquired volunteers using the 3-echo protocol, only the first 3 echoes were considered during training.

All samples were labeled with graph cut results obtained from 6-echo images. Both MR images and labels were masked using thresholding over the mean of all-echoes magnitude images for noisy background removal. To set the R2* and Δ*f* values in the range of [0,1], we normalized the reference variables by 200 [s^−1^] and 400 [Hz], respectively.

The training process elapsed 120 epochs, considering a batch size of 32 and an initial learning rate of 5 × 10^–4^ with a cosine decay. The considered loss function was the mean-absolute-error (MAE) to reference results. We also trained a U-Net (similar implementation of [12]) under the same conditions and hyperparameters configuration, which computed a 4-channel output, each of them corresponding to *ρ*_*w*_, *ρ*_*f*_, R2*, and Δ*f*. Similar to MDWF-Net, U-Net weights were randomly initialized and posteriorly fitted considering the same training samples.

The pre-processing of the data (DICOM reading, rescaling, and graph cut estimations) was performed in MATLAB (MathWorks), and DL-based models were implemented using the TensorFlow framework [27]. Further details about the training process can be found in the Supplementary Material.

### Subjective analysis

*PDFF* maps were visually rated blinded and independently by two experienced radiologists (C.B., J.U., with 10 and 3 years of experience in Body MR, respectively), for a qualitative comparison with the reference method. Complete multi-slice abdomen *PDFF* maps, provided in DICOM format, were assessed in terms of overall image quality, perceived SNR, and artifacts using a 5-point Likert scale (Table 2).

**Table 2:** Scoring criteria used for evaluation of PDFF maps. (*) Types of artifacts include wave artifacts due to inhomogeneities, swapping stains, blurring, and unclear edges, among others

Score	Overall image quality	Signal-to-noise ratio	Artifacts
1	Non-diagnostic	All structures appear to be too noisy	Major artifacts exist and the images are not clinically useful with uninterpretable maps
2	Poor	Most structures appear to be too noisy	Major artifacts exist and clinical use is, therefore, not advised
3	Diagnostic	Some structures appear to be too noisy	Borderline clinical use due to the image quality, with mild or residual artifacts
4	Good	Few structures appear to be too noisy	Minor artifacts which do not adversely affect the clinical use
5	Excellent	There is no noticeable noise on the image	No artifacts and neglectable blurring

^*^ Types of artifacts include wave artifacts due to inhomogeneities, swapping stains, blurring, and unclear edges, among others

### Quantitative and statistical analysis

**Fig. 2:**
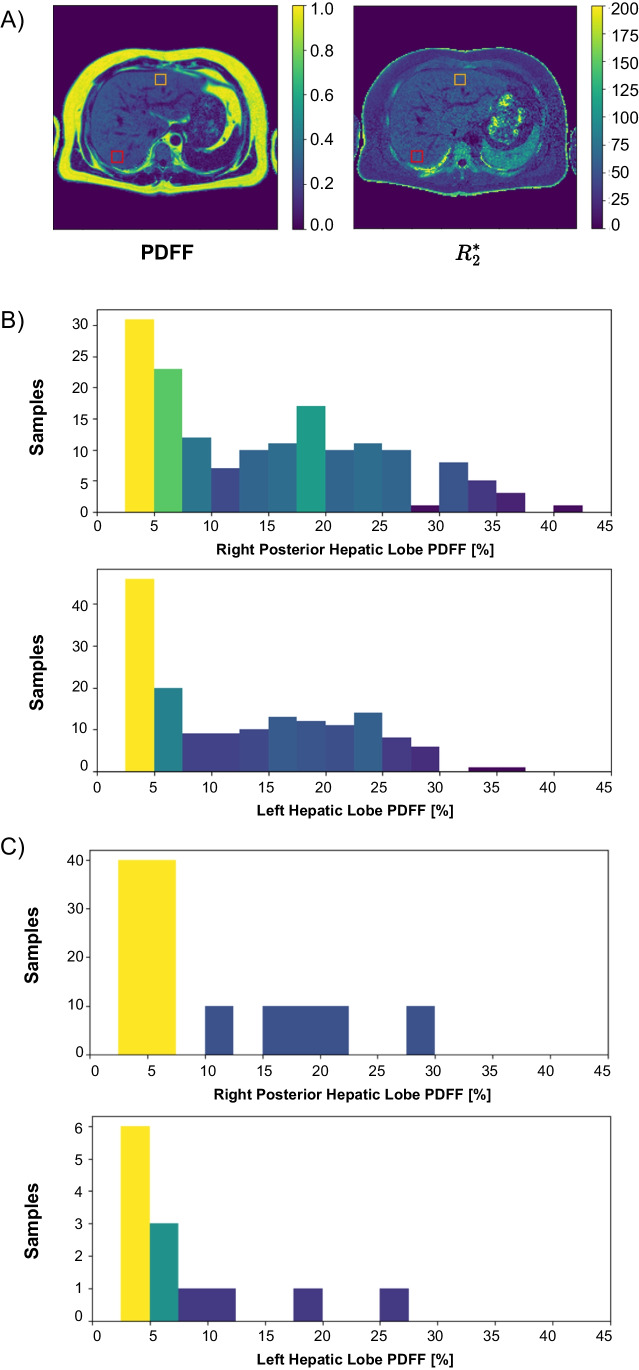
**A** Example of manually located Regions of Interest (ROI) over the right posterior (red) and left (orange) hepatic lobes. **B** Distribution of the reference PDFF values for previously acquired subjects. **C** Distribution of reference *PDFF* for the subjects acquired for this study

To quantitatively assess *PDFF* estimation performance, we located two specific regions of interest (ROI) of ~ 2 cm^2^ in the liver parenchyma, at the right posterior and left hepatic lobes (RHL and LHL, respectively) at the level of the portal bifurcation (Fig. 2A). ROIs were drawn with the assistance of evaluating radiologists, avoiding large vessels, liver lesions, and artifacts, at the same position in the liver. Once the location of each ROI was determined, all of them were co-localized on the maps obtained with each method.

Posteriorly, to assess the bias and blurring of each method, we computed the mean and standard deviation (STD) of *PDFF* at each ROI. For assessing agreement with ground truth (6-echoes graph cuts), we performed least squares (LS) regression correlation and Bland Altman analysis for mean *PDFF* values. Additionally, an ANOVA post hoc test was performed to assess differences between *PDFF* STD values. For this statistical analysis, both ROIs were analyzed together, due to the reduced number of samples. A significance level of 0.05 was considered, and all statistical tests were performed in R (R Development Core Team; https://www.R-project.org). Assessment of R2* maps can be found in the Supplementary Material.

A repository with all the used codes is available at https://github.com/jpmeneses/MDWF-Net.

## Results

### Demographics of the considered subjects

Previously acquired 134 subjects showed the following demographics: mean age, 32.5 ± 18.1 years old; inter-quartile range, 36 years old; 99 women. This first database covered a wide range of body mass index (30.0 ± 5.0 kg/m^2^) and distribution of ground-truth *PDFF* values at RHL and LHL (Fig. 2B).

Additionally, after discarding a single subject due to the presence of several liver cysts, we scanned 13 subjects (mean age, 34.4 ± 13.8 years old; inter-quartile range, 11 years old; 5 women) with both original and 3-echoes protocol (*N* = 290). Although the mean body mass index was lower than the training dataset (25.4 ± 2.2 kg/m^2^), we observed three volunteers whose reference liver *PDFF* was over 20%.

A detailed description of the demographics of both datasets is shown in Table 3.

**Table 3:** Demographics of the patients considered for this study

Attribute	Number or median (with percentiles 25 and 75)		Range	
Subset	Training (only 6 echoes)	Testing (6 and 3 echoes)	Training (only 6 echoes)	Testing (6 and 3 echoes)
Female/male	94/64	5/8	-	-
Age (years old)	26 (16–52)	14–70	28 (26–37)	21–59
Body mass index (kg/m^2^)	30.1 (26.7–34.1)	17.9–40.5	25.6 (23.9–27.5)	21.8–28.9
Reference *PDFF* at RHL (%)	23.3 (14.4–29.8)	7.3–36.1	6.0 (5.0–15.2)	4.2–29.1
Reference *PDFF* at LHL (%)	22.5 (17.8–25.5)	4.2–30.7	5.7 (4.2–8.1)	4.0–25.1

### Computation times

Both CNNs were trained using a Quadro RTX 8000 graphics processing unit (GPU) and tested in a smaller GPU (GeForce GTX 1050) to consider more accessible hardware conditions. Both training processes elapsed ~ 5 h. Once they were trained, mean computation times were 0.07 [s/slice] for U-Net, and 0.13 [s/slice] for VET-Net, compared to 15.58 [s/slice] for 6-echo graph cuts.

### Qualitative analysis

**Fig. 3:**
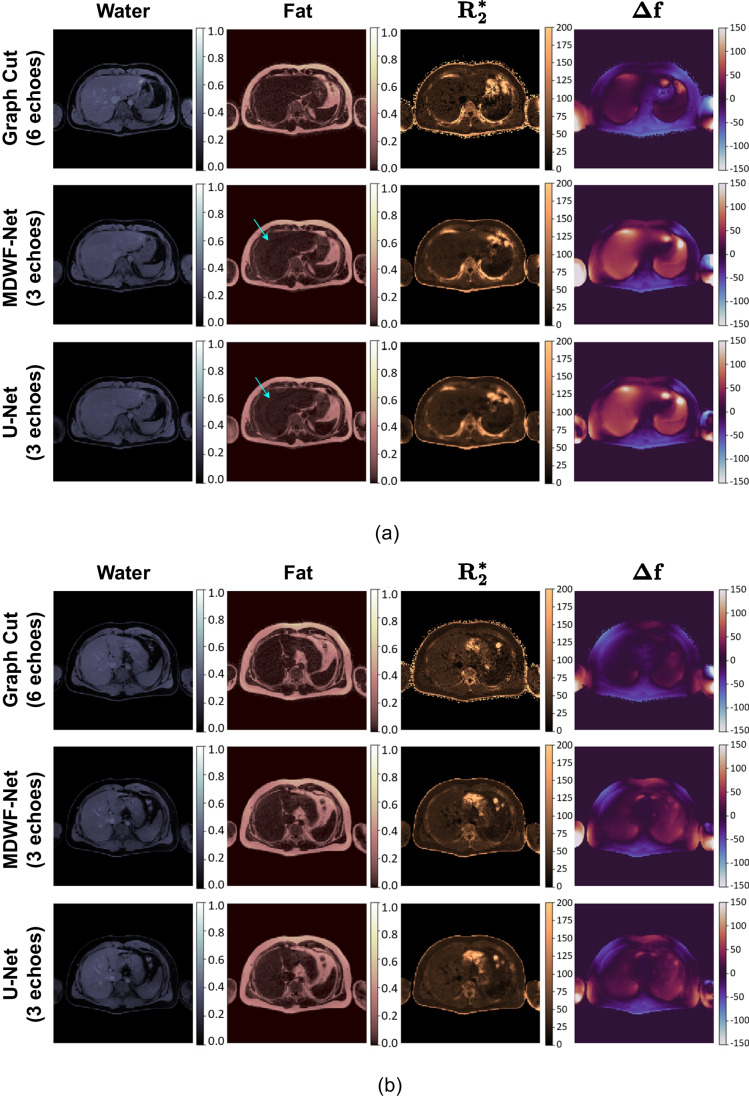
Outputs (*ρ*_*W*_, *ρ*_*F*_, R2*, Δ*f*) for a specific testing sample, obtained using graph cuts (6 echoes), MDWF-Net, and U-Net. **a** Example with swapping artifacts (arrows) for U-Net results. **b** Example for 3-echoes image with a shorter TR (13 ms) than the original protocol (30 ms)

In Fig. 3, we displayed the outputs (*ρ*_*w*_, *ρ*_*f*_, R2*, Δ*f*) for representative slices of testing subjects that were obtained with the graph cuts (using CSE-MR images obtained with the original protocol), and DL-based methods (using 3-echoes images). Figure 3A showed a specific case in which some swapping artifacts were observed in the left anterior part of the liver. In Fig. 3B, we displayed a case in which the TR of the 3-echo pulse sequence was shorter (13.3 ms) than in the original protocol (30.0 ms).

Assessment of radiologists indicated that MDWF-Net and U-Net achieved PDFF maps of similar quality to graph cuts, according to overall IQ scores (U-Net: 3.44 ± 0.80, MDWF-Net: 3.54 ± 0.86, graph cuts: 3.55 ± 0.98). In terms of SNR, both CNNs also achieved similar performance to 6-echo graph cuts (U-Net: 3.27 ± 0.99, MDWF-Net: 3.32 ± 0.76, graph cuts: 3.30 ± 0.88). Moreover, MDWF-Net achieved slightly higher mean artifact reduction scores than 6-echo graph cuts (U-Net: 3.68 ± 0.61; MDWF-Net: 3.66 ± 0.61; graph cuts: 3.56 ± 0.66). All mean *PDFF* scores are summarized in Table 4.

**Table 4:** Mean scores (± standard deviation) assigned to each method in the subjective analysis of resulting PDFF maps. Scores of the two evaluators were treated independently: as there were 28 testing samples, we considered 56 scores for each method

Method	Overall IQ	SNR	Abdominal organs	Hepatic vessels	Artifacts
Graph cuts (6 echoes)	3.55 ± 0.98	3.30 ± 0.88	4.87 ± 0.34	3.98 ± 1.29	3.56 ± 0.66
MDWF-Net (3 echoes)	3.54 ± 0.86	3.32 ± 0.76	4.91 ± 0.28	4.19 ± 1.01	3.68 ± 0.61
U-Net (3 echoes)	3.44 ± 0.80	3.27 ± 0.66	4.91 ± 0.28	4.09 ± 1.04	3.66 ± 0.61

**Fig. 4:**
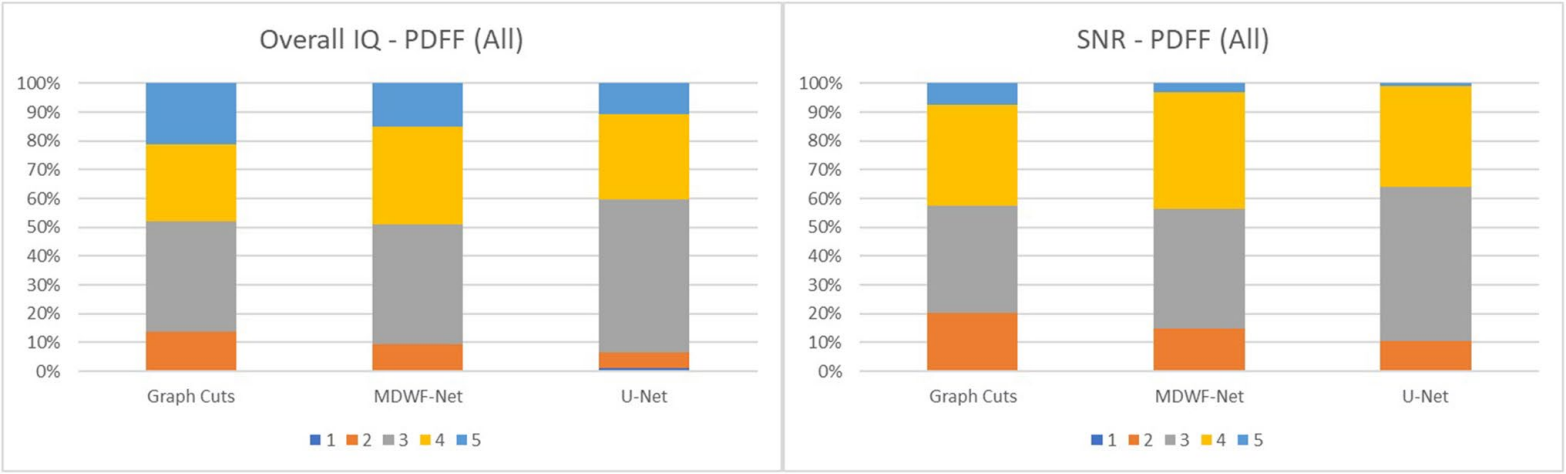
Distribution of overall IQ and SNR scores assigned by the expert radiologists. Results obtained with graph cuts (using 6-echoes images), MDWF-Net, and U-Net (using 3-echo images) were evaluated

The distribution of *PDFF* overall IQ scores showed that almost half of the *PDFF* maps obtained with MDWF-Net were graded with overall IQ scores ≥ 4, unlike U-Net, for which more than half of the samples had a score of 3 (Fig. 4).

### ROI analysis and statistical tests

**Fig. 5:**
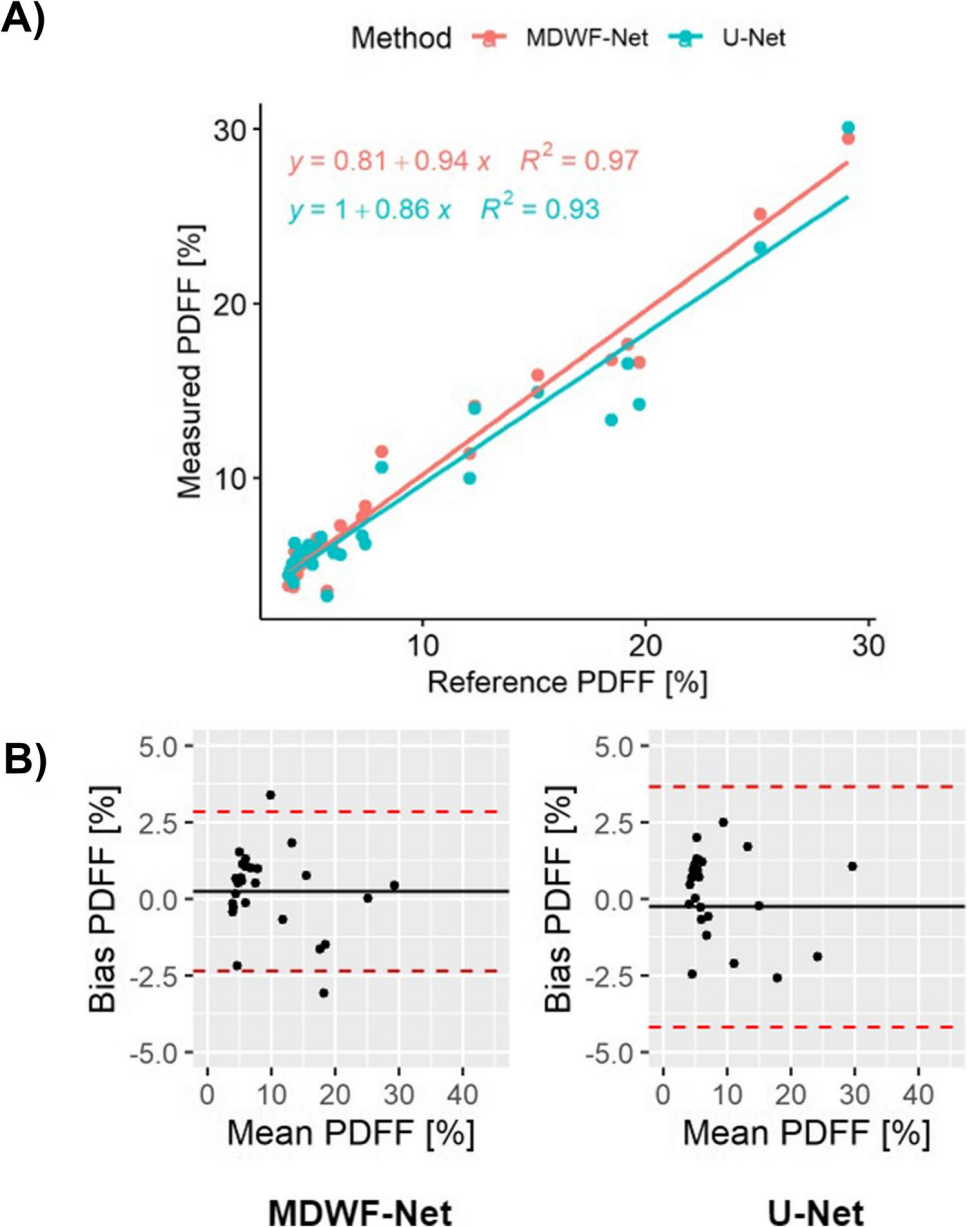
**A**, **B** Statistical analysis of DL-based methods. Least squares regression correlation and Bland Altman analysis were performed for *PDFF* estimations at ROIs

Least squares regression correlation analysis of mean *PDFF* values at ROIs indicated that MDWF-Net showed a better agreement with respect to 6-echo graph cuts (regression slope: 0.94, *R*^*2*^: 0.97) than U-Net (regression slope: 0.86, *R*^2^: 0.93) (Fig. 5). Similarly, Bland Altman analysis showed that MDWF-Net presented a lower bias and narrower LoA (bias: 0.24%; LoA: − 2.36%,2.84%) than U-Net (bias: − 0.27%; LoA: − 4.20%,3.66%).

In the specific case of a short TR subject (same as Fig. 3B), MDWF-Net showed a positive bias of 1.53% and 1.13% in RHL and LHL, while U-Net displayed a bias of 2.00% and 0.94%, respectively.

**Fig. 6:**
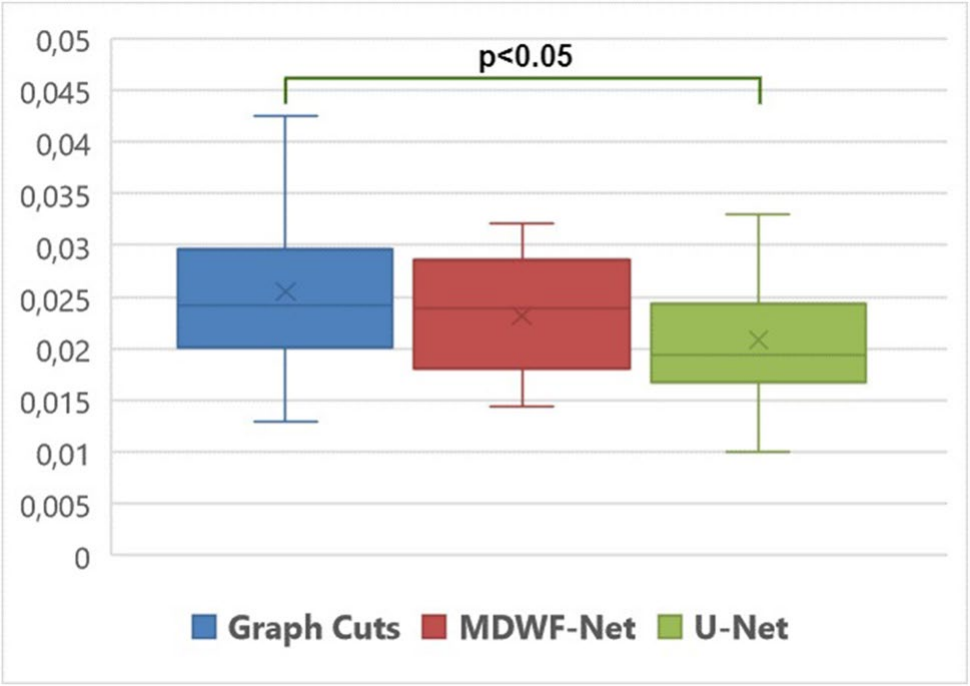
Distribution of standard deviations (STD) of reference 6-echoes Graph Cuts (blue), MDWF-Net (red), and U-Net (green). ANOVA post hoc statistical analysis showed that there were significant differences between ground truth and U-Net

To assess the blurring of *PDFF* estimations, the ANOVA post hoc test considering *PDFF*-STD values at each ROI suggested that there was a significant difference between U-Net and ground-truth (*p* = 0.0250), unlike MDWF-Net, which has no significative difference with respect to the reference (*p* = 0.5323) (Fig. 6).

## Discussion

In this study, we compared the performance of two DL-based methods, our proposed MDWF-Net and a U-Net, an architecture whose performance in MR water-fat separation had been already studied in the literature. For a fair comparison, both CNNs were trained under the same conditions (equal training datasets, same hyperparameters).

Visual assessment of the CNNs outputs showed that, unlike U-Net, MDWF-Net could alleviate some swapping artifacts observed in Fig. 3A. However, we also noticed that both DL-based R2* maps were more blurred than the reference, which was evident in the vascular liver structures. This was expectable, as for most water-fat separation techniques, R2* is the most affected variable when the number of echoes is reduced, and usually up to 12 echoes are suggested for a reliable R2* estimation [10]. In Fig. 3B, we observed that there was no T1-weighting effect when the TR of 3-echo pulse sequence was shorter (13.3 ms) than in the original protocol (30.0 ms).

The reader should notice that we did not quantitatively assess overall maps, such as the ones displayed in Fig. 3, because reference and MDWF-Net results originated from MR images acquired with different protocols and in different instances, which may produce different features (structures, field inhomogeneities, motion, etc.). Therefore, we used a hold-out validation scheme and not a k-fold cross-validation as usually performed in these studies. To quantitatively assess differences, MAE between MDWF-Net and U-Net results, which were obtained from the same 3-echo protocol, was included in Supplementary Material.

Evaluation of radiologists showed that both MDWF-Net and U-Net slightly overperformed the reference method in terms of artifact reduction and perceived SNR scores. Similarly, MDWF-Net showed a visual quality comparable to graph cuts, as demonstrated by the mean overall scores, although our proposed method used less information.

The quantitative assessment showed that MDWF-Net overperformed U-Net in *PDFF* estimation, as showed by the higher correlation with respect to 6-echo graph cut estimations. MDWF-Net also achieved a reduced bias and a narrower LoA than U-Net. Moreover, and in agreement with radiologists’ assessment, MDWF-Net reduced blurring artifacts compared to U-Net, as demonstrated by the ANOVA post hoc analysis. This behavior was expectable, as loss in texture details has been previously reported for U-Net [14].

We believe that the improved *PDFF* estimation of MDWF-Net was a consequence of multi-task architecture, in which the encoder weights (used to compute the shared encoder features) were adjusted considering the decoder weights of each output. As each output, water/fat images, R2*, Δ*f*, has a different range and meaning, the use of separated decoders in MDWF-Net improved *PDFF* estimation compared to U-Net, due to an improved estimation of R2* and Δ*f* effects.

Additionally, we observed that a reduced TR in 3-echo protocol did not significantly affect MDWF-Net performance, probably due to the use of small flip angles [17]. Nevertheless, a limitation of this study was the reduced number of subjects acquired with a shorter TR than the standard protocol. Future work will thoroughly assess T1-weighting bias in a larger group of subjects with few echoes, short TR, and variable flip angles.

Although a quantitative comparison of R2* and Δ*f* between both CNNs was included in Supplementary Material, further validation is part of our future work, as both values could have clinical value. While R2* is potentially useful to diagnose hepatic iron overload [28], Δ*f* maps are used to estimate quantitative susceptibility mapping (QSM) [29, 30]. Nevertheless, this latter application would be necessary to use 3D CSE-MR images.

Incoming work will be focused on adequately assessing the robustness of the proposed method, which is a relevant aspect of translation to clinical practice. MDWF-Net needs to be validated using CSE-MR images of phantoms and subjects acquired using scanners from different vendors.

## Conclusion

MDWF-Net enables a highly accurate estimation of liver *PDFF*, with a performance comparable to graph cuts, using half of the data necessary for the latter technique. This induces a significant shortening of the acquisition time, and therefore a reduction of the breath-holds necessary during the procedure. The favorable assessment of expert radiologists and the absence of statistically significant differences in gold standards demonstrated that the proposed method is a reliable liver fat quantification tool for clinical use.

## Supplementary Information

Below is the link to the electronic supplementary material.

Supplementary file1 (PDF 388 KB)

## Abbreviations

CNN: Convolutional neural network
CSE: Chemical shift-encoded
DL: Deep learning
GRE: Gradient-echo
LHL: Left hepatic lobe
MAE: Mean-absolute-error
MDWF-Net: Multi-decoder water-fat separation network
NAFLD: Non-alcoholic fatty liver disease
PDFF: Proton density fat fraction
RHL: Right hepatic lobe
TR: Repetition time

## Declarations

### Guarantor

The scientific guarantor of this publication is Sergio Uribe.

### Statistics and biometry

One of the authors has significant statistical expertise.

### Study subjects or cohorts overlap

Some study subjects or cohorts have been previously reported in Verdejo et al, Similar Metabolic Health in Overweight/Obese Individuals with Contrasting Metabolic Flexibility to an Oral Glucose Tolerance Test (Frontiers in Nutrition, 2021).

In this prior study, that included J.E.G. as co-author, there were reported on 22 subjects of the current work. The prior report compared metabolic health in individuals with contrasting metabolic flexibility to an oral glucose tolerance test. The current study considered the MR data acquired in this previous work for monitoring abdominal fat.

### Methodology

• Retrospective

• Observational study

• Performed at one institution
