# Supplementary material for: Liver PDFF estimation using a multi-decoder water-fat separation neural network with a reduced number of echoes
Source: Eur Radiol. 2023 Apr 4;33(9):6557–68. doi: 10.1007/s00330-023-09576-2 (PMC10415440; doi:10.1007/s00330-023-09576-2)

Supplementary Material

Training configuration

Training of a neural network consists of an optimization process that adjust the network weights (i.e.: weights on the 2D convolution kernels) in a way that the model learns to solve the problem of interest by iteratively decreasing the value of a loss function. The loss function quantifies the performance that the model is achieving with a specific set of weights. In this case, the loss function was the error of the trained neural network estimations against ground truth.

Particularly, we considered the mean absolute error (MAE) between MDWF-Net ($\widetilde{\Psi}(I_n, \Theta)$), that depends on the input multi-echo image (I_n) and the CNN weights (Θ), and Graph Cuts (Ψ_{ref}) results:

$$Loss = \left|\Psi_{ref} - \widetilde{\Psi}(I_n, \Theta)\right|$$

For a complete reproducibility of the proposed MDWF-Net, the training hyperparameters, which are variables of decision typically involved in training process, were set as follows: Adam optimizer ($\beta_1 = 0.9$, $\beta_2 = 0.999$), cosine learning rate decay starting at 0.0005, 120 epochs, batch size of 32. Data augmentation was performed over the training data, to prevent overfitting of the developed networks. This data augmentation included vertical and horizontal reflections and vertical translations in the range of ±25 pixels.

MDWF-Net converged rapidly, with a MAE plateauing for both training and validation data after the first 40 training epochs. In Figure A1, MAEs of water-fat images, and *R2** and Δf maps are shown.

R2 and Δf estimation performance*

We performed Qualitative and ROI statistical analysis of this biomarker, similar to what we performed to evaluate PDFF estimation performance.

First, evaluation of radiologists indicated that there was a significant diminishing in overall quality of both MDWF-Net and U-Net *R2** maps, compared to reference 6-echoes Graph Cuts method. Moreover, the SNR scores were also lower, as summarized in Figure 2A. ROI statistical analysis displayed a similar trend, as the correlation slope between MDWF-Net and Graph Cuts *R2** measurements at ROIs was of 0.61 (R^2=0.79), higher than U-Net correlation

slope of 0.54 (R^2=0.73). Therefore, MDWF-Net performed better than 3-echoes U-Net, but with non-negligible differences to the Graph Cuts *R2** results. This behavior was expectable, as *R2** quantification has demonstrated to be strongly dependent on the acquired number of echoes. Additionally, we also computed the MAE between MDWF-Net and U-Net results for the testing data: 1) Water/fat: $(2.90 \pm 0.70) \times 10^{-2}$; *R2** (normalized): $(5.08 \pm 1.53) \times 10^{-2}$; Δf (normalized): $(4.95 \pm 1.67) \times 10^{-2}$. These metrics showed that there were significant differences between the estimations of both DL-based models.

Figure A1. Training (blue) and validation (orange) curves of water/fat, R2* and Δf loss functions (mean absolute error with respect to ground truth) during MDWF-Net weight fitting.

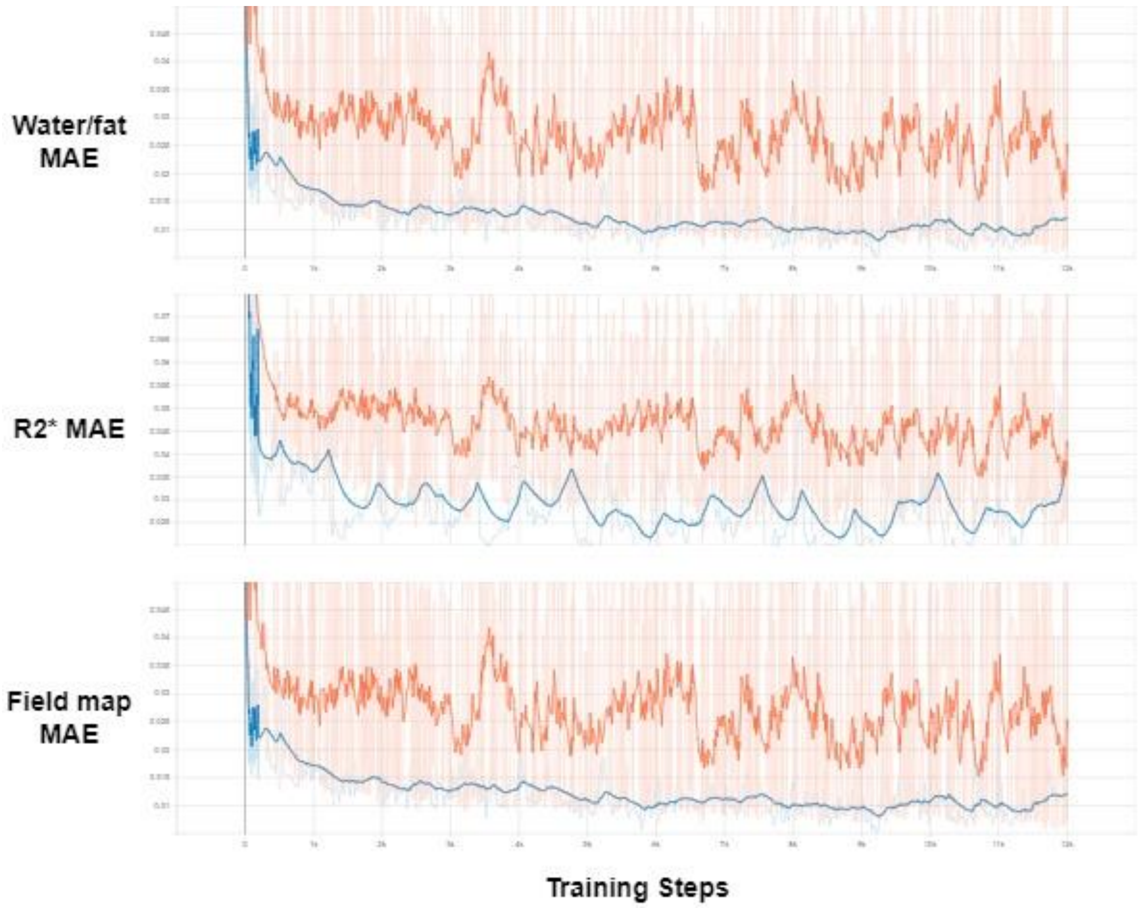

Figure A2. Distribution of Overall IQ and SNR scores assigned to R2* maps by the expert radiologists. Results obtained with Graph Cuts (using 6-echoes images), MDWF-Net, and U-Net (using 3-echoes images) were evaluated.

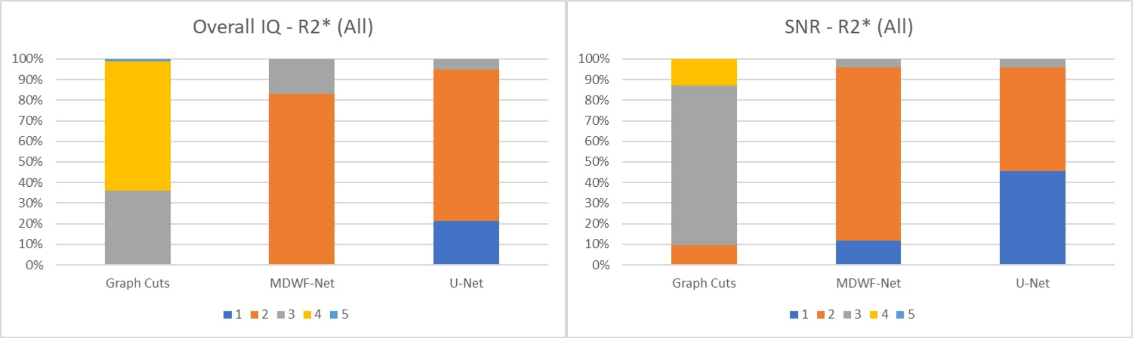

Figure A3. Statistical analysis of R2* measurements at ROIs obtained with DL-based methods. Least squares regression correlation and Bland Altman analysis were performed.

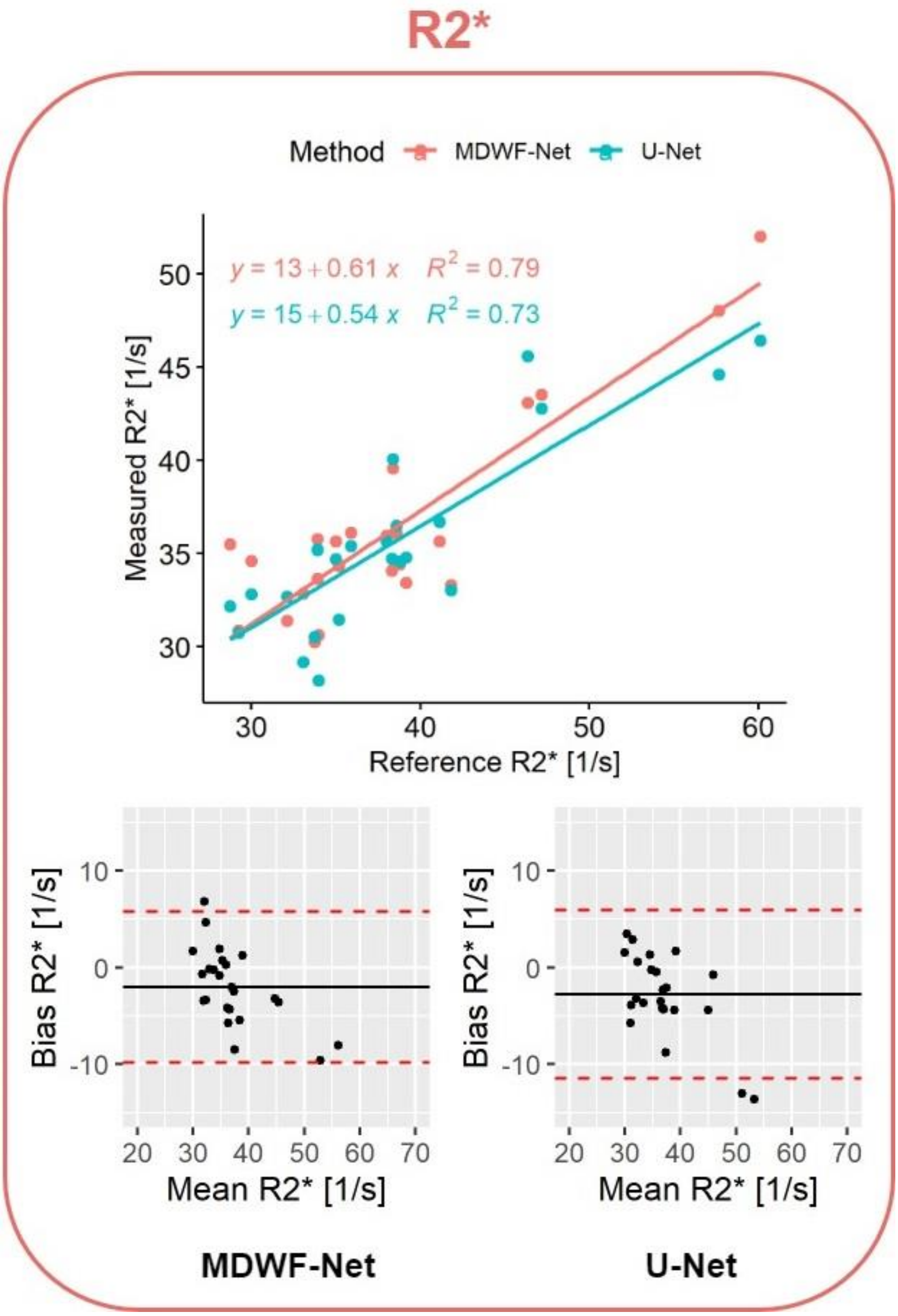

Supplement: Supplementary file 1 — Supplementary file1 (PDF 388 KB) [file 330_2023_9576_MOESM1_ESM.pdf]
